# Visceral Adiposity Index (VAI) in Children and Adolescents with Obesity: No Association with Daily Energy Intake but Promising Tool to Identify Metabolic Syndrome (MetS)

**DOI:** 10.3390/nu13020413

**Published:** 2021-01-28

**Authors:** Sara Vizzuso, Alberico Del Torto, Dario Dilillo, Valeria Calcaterra, Elisabetta Di Profio, Alessandro Leone, Luisa Gilardini, Simona Bertoli, Alberto Battezzati, Gian Vincenzo Zuccotti, Elvira Verduci

**Affiliations:** 1 Department of Pediatrics, Vittore Buzzi Children’s Hospital, University of Milan, 20154 Milan, Italy; dario.dilillo@asst-fbf-sacco.it (D.D.); valeria.calcaterra@unipv.it (V.C.); gianvincenzo.zuccotti@unimi.it (G.V.Z.); elvira.verduci@unimi.it (E.V.); 2 Centro Cardiologico Monzino IRCCS, 20138 Milan, Italy; alberico.deltorto@cardiologicomonzino.it; 3 Pediatric and Adolescent Unit, Department of Internal Medicine, University of Pavia, 27100 Pavia, Italy; 4 Science Nutrition, University of Milan, 20154 Milan, Italy; elisabetta.diprofio@unimi.it; 5 International Center for the Assessment of Nutritional Status (ICANS), Department of Food, Environmental and Nutritional Sciences (DeFENS), University of Milan, Via Sandro Botticelli 21, 20133 Milan, Italy; alessandro.leone1@unimi.it (A.L.); simona.bertoli@unimi.it (S.B.); alberto.battezzati@unimi.it (A.B.); 6 Istituto Auxologico Italiano, IRCCS, Lab of Nutrition and Obesity Research, 20145 Milan, Italy; l.gilardini@auxologico.it; 7 Department of Health Sciences, University of Milan, 20154 Milan, Italy

**Keywords:** visceral adiposity index, pediatric obesity, metabolic syndrome

## Abstract

(1) Background. Visceral adiposity index (VAI) has been recently identified as a new cardiometabolic risk marker reflecting abdominal fat distribution and dyslipidaemia. The aim of the present paper was to evaluate the relationship between VAI, daily energy intake and metabolic syndrome (MetS) in a cohort of obese Caucasian children and adolescents, aged 8 to 15 years. (2) Methods. Consecutive Italian children and adolescents with obesity, according to World Health Organization were enrolled. Anthropometric parameters and blood pressure were measured. Fasting blood samples have been analyzed for lipids, insulin and glucose levels. MetS was diagnosed using identification and prevention of dietary- and lifestyle-induced health effects in children and infants (IDEFICS) or International Diabetes Federation (IDF) criteria according to age. Homeostatic model assessment index (HOMA-IR), quantitative insulin sensitivity check index (QUICKI), A body shape index (ABSI) and VAI were calculated. Multivariable logistic regression analyses with sex, age and each anthropometric parameter (body mass index (BMI) z-score, ABSI, waist-to-height ratio (WHR)) or VAI was performed to predict MetS. Receiver operation curve (ROC) analysis was used to define the optimal VAI cut-off to identify MetS. Multiple regression was performed to predict the BMI z-score and VAI from daily energy intake after adjusting for age and sex. (3) Results. Six hundred and thirty-seven (313 boys and 324 girls) children and adolescents with obesity with median age 11 (interquartile range 10–13) years were included in the analysis. MetS was diagnosed in 79 patients. VAI correlated with BMI, WHR, ABSI, HOMA-IR, QUICKI, systolic blood pressure, low- and high-density lipoprotein cholesterol, triglycerides and triglycerides-to-HDL ratio (*p* < 0.050). Optimal VAI cut-off (AUC) values to identify MetS were 1.775 (0.774), 1.685 (0.776) and 1.875 (0.797) in the whole population, boys and girls, respectively. Energy intake was positively associated with BMI z-score but no association was found with VAI. (4) Conclusion. VAI is a promising tool to identify MetS in children and adolescents with obesity and should be used in the management of abdominal obesity together with dietary assessment.

## 1. Introduction

In terms of prevalence and economic significance [1,2] pediatric obesity is considered one of the most important public health problems of the 21st century [3]. Both during childhood and adolescence, children with obesity can often present glucose metabolism disorders such as insulin resistance, also dyslipidemia or hypertension, all classic signs of the metabolic syndrome (MetS) [4,5]. Most of these metabolic disorders are driven by excess central (intra-abdominal) body fat distribution [6]. It is well recognized that behavioral changes and lifestyle modifications, including dietary habits, are essential to prevent and manage childhood obesity [7,8,9,10].

In epidemiological studies and clinical settings, many anthropometric indices reflecting general and abdominal obesity, have been proposed. Body mass index (BMI) is the most frequently used index; it is a substitute for body composition assessment [11], which as a limitation in the impossibility to distinguish lean mass from fat mass and its distribution [12]. Accordingly, the use of age- and sex-adjusted BMI z-score has been recommended in pediatric age instead of BMI alone; however, the association between cardio metabolic-risk and pediatric BMI z-score is not linear [13].

Other indexes that could be more predictive in identifying the metabolic syndrome have been evaluated. Waist circumference (WC), which also reflects the distribution and percentage of body fat, has been studied to assess body composition and cardio-metabolic risk [14]. Studies showed that WC is more predictive than BMI for hypertension and impaired glucose metabolism [15,16].

An index that offers more advantages than BMI and WC is the waist to height ratio (WHR) [17] and it has, therefore, been suggested as a good predictor of MetS in pediatric age [18]. During routine outpatient evaluation, it was suggested by Joyce et al., to use WHR as a screening measure to identify adolescent with high risk for hypertension [19]. Even though several studies have been unable to demonstrate a significant difference in predicting cardio-metabolic risks for the above-mentioned indices [20,21,22].

In addition, A body shape index (ABSI) has been validated as an index related to abdominal and peripheral fat [23]. It further underlines the critical relationship between metabolic and cardiovascular alterations and waist circumference in obesity [24,25]. In the pediatric population of children with obesity and overweight, ABSI has been shown to have significant associations with in cardiometabolic risk markers [26,27].

Visceral adiposity index (VAI) has recently been identified as a new cardio-metabolic risk marker as it reflects abdominal fat distribution and dyslipidemia. It has already been shown to be associated with resistance to insulin action, abnormalities in glucose balance and an increased risk of cardiovascular disease in adults [28,29,30]. This index is calculated according to a sex-specific mathematical model that relates some anthropometric measures (BMI and WC) to some laboratory parameters (triglycerides (TG) and high-density lipoprotein cholesterol (HDL-C)) [31]. Furthermore, VAI index is also a useful tool for detecting MetS in children and adolescent [32].

However, a universally recognized reference value for VAI predictive of increased cardio-metabolic risk has not been determined to date in the pediatric population. In 2019, Ejtahed et al. published a cross-sectional study conducted in a population of 3843 Iranian students aged 7 to 18 years with the aim of obtaining cut-off values for VAI to assess its relationship with MetS [32]. The cut-offs identified for VAI in predicting MetS were 1.58, 1.30 and 1.78 in the total population, boys and girls, respectively. In this age group, VAI has been shown to be associated with cardio-metabolic risk factors such as visceral obesity, altered fasting blood glucose (IFG), reduced HDL-C and increased low-density lipoprotein cholesterol (LDL-C); therefore, VAI can be used as a surrogate marker of visceral adiposity and a good predictor of MetS in pediatric age.

Moreover, a study evaluated the association between dietary macronutrient proportions and prospective VAI changes in an adult population and demonstrated that a higher dietary proportion of protein and animal-derived monounsaturated fatty acids may be positively associated with VAI changes and risk of visceral adiposity dysfunction [33]. Nevertheless, to date, there are no studies correlating energy intake and VAI conducted on children and adolescents.

The primary aim of this cross-sectional study was to evaluate the relationship between the anthropometric index VAI, daily energy intake and MetS in a cohort of obese Caucasian children and adolescents, aged 8 to 15 years. A secondary aim was to identify which of different anthropometric adiposity indexes allows a better assessment of the probability of having MetS.

## 2. Materials and Methods

### 2.1. Cohort

We performed an observational cross-sectional study. Consecutive Caucasian children and adolescents, diagnosed as obese according to World Health Organization (WHO) criteria [34], aged 8–15 years, recruited at V. Buzzi Children’s Hospital in Milan (Italy), International Center for the Assessment of Nutritional Status (ICANS), University of Milan and Istituto Auxologico Italiano, IRCCS, Lab of Nutrition and Obesity Research in Milan, between January 2014 and January 2019, have been enrolled.

We excluded children and adolescents affected by genetic or syndromic obesity (e.g., Prader Willi syndrome, Bardet–Biedl syndrome and genes related to the leptin–melanocortin axis) or by hormonal conditions (e.g., Cushing’s syndrome, hypothyroidism, growth factor deficiency and congenital hyperinsulinism) [8] besides obesity, on use of antihypertensive, antidiabetic or lipid-lowering medication and/or medication that could influence body weight. The study was conducted in accordance with the local medical ethical committee (protocol number 2015/ST/135). Written informed consent was given by a parent for all enrolled subjects. On the same morning, the enrolled subjects underwent a medical interview, an anthropometric assessment (with detection of BMI, ABSI, WHR and VAI), a measurement of systolic blood pressure (SBP) and diastolic (DBP), and a blood sample.

### 2.2. Measurements

#### 2.2.1. Anthropometry

Weight and height were assessed applying a medical-certified scale and children’s medical-certified stadiometer, respectively following international guidelines [35]. BMI was calculated as [36]:

$$\mathrm{BMI}=\frac{\mathrm{Weight}\;\left(\mathrm{kg}\right)}{\mathrm{Height}\;{\left(m\right)}^{2}}$$

BMI values were transformed into related z-scores using the WHO reference growth charts for age and sex [34]. Obesity was defined as BMI z-score ≥2. Waist circumference was measured trough an inextensible anthropometric tape positioned parallel to the floor, at midpoint between costal margin and iliac crest, in a standing position, at the end of a quiet expiration [35].

Fat mass (FM), FM percentage (FM%), fat-free mass (FFM) and fat-free mass percentage (FFM%) were estimated using a bioelectrical impedance analysis system (BC 418 MA, Tanita Corp, Nutrients 2020, 12, 1785 3 of 13 Tokyo, Japan [37]. An oscillometer device was used to check blood pressure (BP), according to the national recommendations [38].

#### 2.2.2. Adiposity Indices

ABSI was calculated according to the following formula [39], rounding BMI to the second decimal place:

$$\mathrm{ABSI}=\frac{\mathrm{WC}\;\left(m\right)}{{\mathrm{BMI}}^{2/3}\times \mathrm{Height}\;m^{1/2}}$$

WHR was calculated as [40]:

$$\mathrm{WHR}=\frac{\mathrm{WC}\;\left(m\right)}{\mathrm{Height}\;\left(m\right)}$$

A WHR value over 0.60 has been recently associated to a higher risk for MetS in children and adolescents [41].

VAI reflects fat distribution and metabolism and is calculated as:

$$\mathrm{VAI}\;\left(\mathrm{males}\right)=\frac{\mathrm{WC}\;}{39.68+\left(1.88\times \mathrm{BMI}\right)}\times \frac{\mathrm{TG}\;}{1.03}\times \frac{1.31}{\mathrm{HDL}-C}$$

$$\mathrm{VAI}\;\left(\mathrm{females}\right)=\frac{\mathrm{WC}\;}{39.58+\left(1.89\times \mathrm{BMI}\right)}\times \frac{\mathrm{TG}}{0.81}\times \frac{1.52}{\mathrm{HDL}-C}$$

WC is measured in centimeters, BMI in Kg/m^2^, TG and HDL-C in mmol/L [29].

#### 2.2.3. Biochemistry

Blood samples were obtained in standardized conditions: From 8:30 to 9:00, after 12 h of fasting for measurement of total cholesterol (TC), HDL-C, LDL-C, TG, insulin and fasting glucose. US National Heart, Lung, and Blood Institute (NHLBI) lipid cutoff values, based on US normative data, were used to detect dyslipidemia [42]. Insulin and fasting glucose, levels were compared to our Clinical Laboratory range values.

#### 2.2.4. Dietary Habits

Subjects’ dietary habits were assessed through a food frequency questionnaire (FFQ) developed in 1990 at Department of Health Sciences, University of Milan, based on the original Block-FFQ [43,44] and revised in 2008 according to the full-length Block 2005 FFQ © (NutritionQuest, Berkeley, CA, USA) and the 2007 new national food composition tables [45]. The FFQ is the most common method for dietary assessment used in large epidemiological studies [46]. The questionnaire consists of a list of 120 foods and beverages with response categories to indicate usual (daily, weekly or monthly) frequency of consumption and portion (full, half or double portion). The questionnaire was administered by dieticians as a face-to-face interview to children (or adolescents) together with their parents. Usual portion sizes were estimated using household measures and the weight (e.g., pasta) or unit (e.g., fruit juice) of the purchase. In addition, a 24 h recall was recorded at the end of the inter-view to standardize the usual serving size. Energy intake analysis was performed using an ad hoc PC software program capable of elaborating diets and analyzed food diaries into macro and micronutrients (MetadietaVR, 2013; METEDAsrl, via S.Pellico 4, San Benedetto del Tronto, AP, Italy).

#### 2.2.5. Metabolic Syndrome

Distinct criteria have been applied for the diagnosis of MetS according to age groups. For children aged from 7 to 10 years, MetS was defined as reported by Ahrens et al. [47] in the identification and prevention of dietary- and lifestyle-induced health effects in children and infants (IDEFICS) study, with at least three of the following criteria: WC ≥90th percentile [48]; SBP or DBP ≥90th percentile by sex and age [49]; TG ≥90th percentile or HDL-C ≤10th percentile by sex and age [50]; homeostatic model assessment for insulin resistance (HOMA-IR) ≥90th percentile or fasting blood glucose ≥90th percentile by sex and age [51]. For children aged from 10 to 16 years, MetS was identify as proposed by the International Diabetes Federation (IDF) recommendations [4], with WC ≥90th percentile byage and sex [52] combined with at least 2 of the following criteria: Fasting blood glucose ≥100 mg/dL (≥5.6 mmol/L); TG ≥150 mg/dL (≥1.7 mmol/L); HDL-C <40 mg/dL; SBP ≥130 mmHg or DBP ≥85 mmHg.

#### 2.2.6. Cardiometabolic Risk Assessment

HOMA-IR index, HOMA of percent β-cell function (HOMA-β) and the quantitative insulin-sensitivity check index (QUICKI) are useful tools in the clinical practice to detect subjects at risk for type 2 diabetes mellitus, especially children and adolescents [53].

The HOMA-IR was calculated using the following formula [54]:

$$\mathrm{HOMA}-IR=\frac{\mathrm{Glucose}\left(\frac{\mathrm{mmol}}{L}\right)\times \;\mathrm{Insulin}\left(\frac{\mathrm{mU}}{\mathrm{mL}}\right)}{22.5}$$

It is the most widely used method to assess the insulin resistance. HOMA-IR changes by age and gender. Recently, HOMA-IR reference values were published for a large population of young, normal weight and obese Caucasians. According to Shashaj et al., a HOMA-IR value ≥75th percentile in obese participants identifies adolescents with cardio-metabolic risk factors [55].

HOMA-β is an index of β-cell function, calculated as [56]:

$$\mathrm{HOMA}-\beta =\frac{20\;\times \;\mathrm{Insulin}\;\left(\frac{\mathrm{mU}}{\mathrm{mL}}\right)\;}{\mathrm{Glucose}\left(\frac{\mathrm{mmol}}{L}\right)-3.5}$$

QUICKI, considered as a surrogate measure of insulin sensitivity [57] was calculated using the following formula:

$$\mathrm{QUICKI}=\frac{1\;}{\log10\;\mathrm{Insulin}\;\left(\frac{\mathrm{mU}}{\mathrm{mL}}\right)+\;\log10\;\mathrm{Glucose}\left(\frac{\mathrm{mg}}{\mathrm{dL}}\right)}$$

considering a reference value of 0.37 ± 0.04 [57,58].

The triglyceride–glucose index (TyG index) mostly indicates muscles’ resistance to insulin action [59] and it is calculated as:

$$\mathrm{TyG}\;\mathrm{index}=\mathrm{Ln}\frac{\left[\mathrm{Triglycerides}\;\left(\frac{\mathrm{mg}}{\mathrm{dL}}\right)\;\times \;\mathrm{Glucose}\;\left(\frac{\mathrm{mg}}{\mathrm{dL}}\right)\right]}{2}$$

Children and adolescents at risk of atherogenic dyslipidemia and impaired fasting glucose (IFG) have a value of TG (mg/dl)/HDL-C (mg/dl) ratio (TG/HDL) ≥2.2 [60,61].

Moreover, VAI index is also a useful tool for detecting MetS in children and adolescent [32].

### 2.3. Statistical Analysis

Shapiro–Wilk test was used to assess normality of each continuous variable. As all tested variables were non-normally distributed, they were summarized with median (interquartile range). Discrete variables were reported as frequency and percentage. Characteristics of patients with and without MetS, aged <10 and ≥10 years, boys and girls, with BMI z-score <3 and ≥ 3, were compared using Mann–Whitney U test. χ^2^ test was used to compare frequencies of discrete variables among different subgroups. Spearman correlation test was used to assess continuous variables correlations. Sex- and age-adjusted logistic multivariable analysis models were used to assess the association between BMI z-score, ABSI z-score, WHR z-score or VAI z-score with MetS. McFadden pseudo-R^2^ was used as a measure of association. Akaike informative criterion (AIC) was used to compare different models: The choice of the best predictive model was based on the lower AIC. Receiver operation curve (ROC) analysis with Youden J statistics was used to identify the optimal VAI cut-off to detect MetS. Multivariable linear regression was performed to predict BMI z-score and VAI (in separate models) from daily energy intake after adjusting for age and sex. *p*-values < 0.050 were considered statistically significant. Statistical analyses were performed using SPSS Statistics version 20 (IBM Corp., Armonk, NY, USA) and R version 4 (R Core Team, Vienna, Austria).

## 3. Results

Six hundred and thirty-seven (313 boys, and 324 girls) children and adolescents with obesity were included in the analysis. Median age was 11 (interquartile range 10–13) years. Anthropometric characteristics, glyco-metabolic and lipid parameters, VAI and MetS prevalence in the whole cohort and in prespecified subpopulations according to sex, age, BMI z-score and presence of MetS are shown in Table 1 and Table 2 and Supplementary Table S1. Boys were taller, had higher BMI z-score and ABSI than girls. Instead, girls had significantly higher HOMA- β and VAI (Table 1).

Among subjects with BMI z-score ≥3 there were more girls; they were significantly younger, shorter, had significantly higher WC, WHR, HOMA-IR, HOMA-β, glycemia and DBP with QUICKI significantly lower (Table 2).

MetS was diagnosed in 79 patients (12.4%); MetS patients were significantly younger and shorter, had higher, HOMA-IR, HOMA-β, TyG index, TC, TG, LDL-C, triglycerides-to-HDL ratio, VAI and SBP, and a lower BMI, WC, HDL-C and QUICKI (*p* < 0.050). BMI z-score, ABSI and WHR in patients with or without MetS did not differ significantly (Table 2).

VAI significantly correlated with BMI, WC, WHR, ABSI, HOMA-IR, HOMA-β, TyG index, QUICKI, TC, TG, HDL-C, LDL-C, triglycerides-to-HDL ratio and SBP (Table 3, Supplementary Table S2).

A logistic multivariable model including sex, age and VAI was the best predictor of MetS when compared to models including sex, age, and BMI z-score or ABSI z-score or WHR z-score (*p* < 0.050, ψR^2^ 0.229) (Table 4).

**Figure 1:**
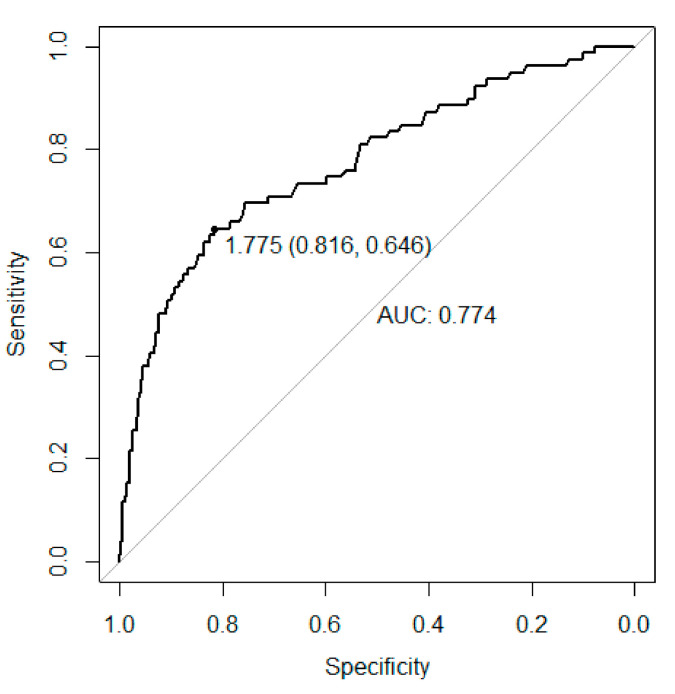
Receiver operation curve (ROC) analysis to find the optimal Visceral Adiposity Index (VAI) cut-off to identify MetS in the whole population.

**Figure 2:**
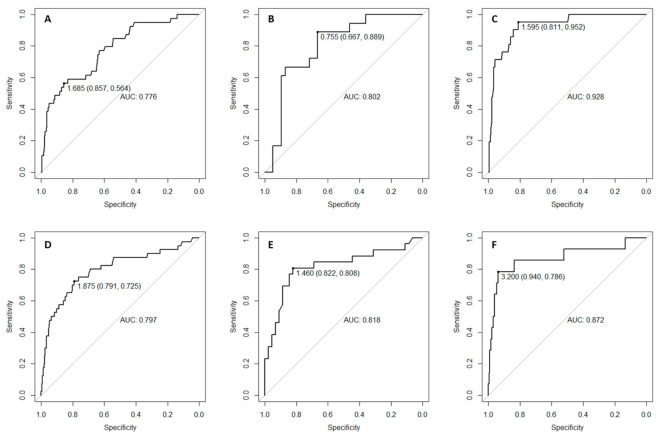
ROC analysis to find the optimal VAI cut-off to identify MetS in boys (**A**), boys aged <10 years (**B**), boys aged ≥10 years (**C**), girls (**D**), girls aged <10 years (**E**) and girls aged ≥10 years (**F**).

ROC analysis identified the optimal VAI cut-off to predict MetS. The optimal cut-off (AUC) was 1.775 (0.7744), 1.685 (0.7761) and 1.875 (0.7968) in the whole population, boys and girls, respectively (Figure 1 and Figure 2).

Energy intake was available in a subset of 272 patients. By multiple regression analysis, a model including energy intake, sex and age was positively associated with BMI z-score (*p* < 0.001) but not with VAI, in the whole cohort and in subgroups by sex and age <10 years and ≥10 years.

## 4. Discussion

In the present study six hundred and thirty-seven children and adolescents with obesity were studied. Seventy-nine patients (12.4%) were diagnosed with MetS in our population. This finding is comparable to the overall prevalence of MetS in other cross-sectional studies conducted in obese pediatric population, with rates ranging from 10% to 38% [62,63,64,65,66]. The real prevalence of this condition in children and adolescents is hard to estimate due to the lack of a consensus on its definition [62,63,64,65], we tested for the first time the relationship between different anthropometric and adiposity indexes, including VAI, and MetS risk in a large sample of Caucasian children and adolescents with obesity, also taking into account the effects of sex and age. BMI z-score, ABSI and WHR were not different in patients with or without MetS. A logistic multivariable model including sex, age and VAI was the best predictor of MetS when compared to models including sex, age, and BMI z-score or ABSI or WHR. Our results are interesting considering that VAI, calculated according to a sex-specific mathematical model that relates some anthropometric measures (BMI and WC) to some laboratory parameters (TG and HDL-C) has been recently presented as a new marker to better define cardiometabolic risk compared to BMI alone, both in children and in adults [28,29,30,32]. We also identified optimal VAI cut-offs to help in diagnosing MetS with high specificity. It is important to note that, as cut-offs vary in relation to sex and age group, it would be better to use sex- and age-corrected cut-offs, as proposed in the results, to identify subjects at higher risk of having MetS. It is also important to note that VAI cut-offs may differ if alternative criteria for MetS diagnosis are used.

Visceral abdominal fat tissue (VAT) has been shown to be fundamental in the pathogenesis of MetS, both in adults and in children [67]. Computed tomography (CT) and magnetic resonance imaging (MRI) are the reference methods for the assessment of VAT, but they cannot be used in routine clinical practice and epidemiological research. VAT by CT and MRI correlation with VAI has never been investigated.

Multiple regression was performed to predict the BMI z-score and VAI from daily energy intake after adjusting for age and sex. Energy intake was positively associated with BMI z-score, but no association was found with VAI. These findings are consistent with a recent our study [68]. To our knowledge no other study has investigated the association between daily energy intake and VAI.

Moreover, in our study a ROC analysis identified the optimal VAI cut-off to identify MetS. The optimal cut-off was 1.775, 1.685 and 1.875 in the whole population, boys and girls, respectively. As our study was conducted in a cohort of children and adolescents with obesity, VAI cut-offs are slightly higher than the ones published by Ejtahed et al. in a cohort of Iranian children and adolescents that included obese and non-obese subjects; as expected VAI cut-offs in our study had also a higher specificity and lower sensitivity than those reported by Ejtahed et al. [32].

The present study has noteworthy strengths. First of all, we studied a large cohort representing a wide range of age of both sexes, contributing to obtaining robust results. Additionally, the studied sample can be considered homogeneous, as participating children and adolescents were from the same geographical region, and shared similar culture, lifestyle, and eating habits.

The study also has potential limitations. Indeed, the sample of Caucasian children and adolescent was self-selected. Our findings are not necessarily applicable to general populations and to other ethnic groups. Therefore, more studies are needed to determine whether the results obtained are consistent in larger samples of children and adolescents with obesity in the same age group.

## 5. Conclusions

In conclusion, VAI is a promising tool to identify MetS in children and adolescents with obesity and should be used in the management of abdominal obesity together with dietary assessment. Further prospective longitudinal studies aiming to evaluate the capability of VAI cut-offs to predict longitudinal outcomes in pediatric population are warranted [66], also including the evaluation of VAT.

## Figures and Tables

**Table 1:** Characteristics of the population according to gender and age group

	Cohort		Boys		Girls			<10 Years		≥10 Years		
	(*n* = 637)		(*n* = 313)		(*n* = 324)		*p*	(*n* = 129)		(*n* = 508)		*p*
Age	11	(10–13)	11	(10–13)	12	(10–13)	0.757	9	(8–9)	12	(11–13)	<0.001
Girls	324	(50.9%)	-		-		-	72	(55.8%)	252	(49.6%)	0.208
Height	1.54	(1.45–1.62)	1.55	(1.45–1.65)	1.53	(1.45–1.60)	0.026	1.4	(1.35–1.44)	1.57	(1.50–1.64)	<0.001
Weight	70	(56.3–83.4)	69.5	(56–85.3)	72.4	(56.4–82.7)	0.968	49.3	(44.6–53.9)	75.8	(63.6–88)	<0.001
BMI	29.3	(26.4–31.9)	28.8	(26.2–31.1)	30	(26.6–32.7)	0.003	25.2	(24–27)	30.2	(27.6–32.7)	<0.001
BMI z-score	2.9	(2.6–3.1)	2.9	(2.6–3.2)	2.8	(2.6–3)	<0.001	3	(2.7–3.3)	2.8	(2.6–3)	0.003
WC	94	(86–102)	94	(87–103)	93	(84–101)	0.052	82	(78–87)	96	(90–104)	<0.001
WHR	0.61	(0.58–0.65)	0.61	(0.58–0.65)	0.62	(0.57–0.65)	0.612	0.59	(0.56–0.64)	0.62	(0.58–0.65)	0.001
ABSI	0.0800	(0.0766–0.0833)	0.0814	(0.0783–0.0837)	0.0785	(0.0747–0.0824)	<0.001	0.0805	(0.0781–0.0839)	0.0798	(0.0759–0.0831)	0.007
Glucose	85	(80–90)	86	(81–90)	85	(80–90)	0.144	83	(79–88)	86	(81–90)	0.002
HOMA-IR	3.22	(2.18–4.72)	3.12	(2.05–4.57)	3.32	(2.39–4.87)	0.050	2.34	(1.61–3.75)	3.47	(2.46–4.9)	<0.001
HOMA β	256.9	(180.0–373.8)	237.9	(163.3–340.6)	271.2	(194.3–420.0)	0.001	222.1	(144.2–315.9)	269.0	(187.6–386.3)	0.001
QUICKI	0.321	(0.305–0.340)	0.322	(0.306–0.342)	0.320	(0.303–0.335)	0.055	0.336	(0.315–0.355)	0.318	(0.303–0.334)	<0.001
TyG index	4.42	(4.25–4.58)	4.43	(4.24–4.58)	4.41	(4.25–4.57)	0.979	4.38	(4.24–4.57)	4.42	(4.27–4.58)	0.155
TG	80	(58–108)	80	(57–109)	79	(61–108)	0.893	76	(57–106)	81	(59–111)	0.306
TC	156	(140–177)	157	(142–180)	155	(138–175)	0.101	155	(142–177)	156	(139–177)	0.996
HDL-C	47	(40–54)	47	(39–54)	47	(40–54)	0.892	48	(42–55)	46	(39–54)	0.038
LDL-C	93	(78–111)	96	(78–113)	92	(78–110)	0.307	94	(80–110)	93	(78–111)	0.939
TG/HDL ratio	1.7	(1.2–2.5)	1.7	(1.1–2.5)	1.7	(1.2–2.5)	0.893	1.6	(1–3.1)	1.8	(1.2–2.6)	0.094
VAI	1.13	(0.75–1.76)	0.95	(0.62–1.47)	1.37	(0.95–1.97)	<0.001	1.02	(0.66–1.62)	1.17	(0.77–1.77)	0.026
SBP	110	(105–120)	111	(105–120)	110	(105–120)	0.455	105	(100–111)	115	(110–120)	<0.001
DBP	70	(60–71)	70	(60–74)	70	(60–71)	0.711	60	(58–70)	70	(62–75)	<0.001
MetS	79	(12.4%)	39	(12.5%)	40	(12.3%)	0.965	44	(34.1%)	35	(6.9%)	<0.001

Body mass index z-score (BMI z-score), waist circumference (WC), waist-to-height ratio (WHR), A body shape index (ABSI), homeostatic model assessment index—insulin resistance (HOMA-IR), homeostatic model assessment index-β (HOMA-β), quantitative insulin sensitivity check index (QUICKI), triglyceride–glucose index (TyG index), triglycerides (TG), total cholesterol (TC), high-density lipoprotein cholesterol (HDL-C), low-density lipoprotein cholesterol (LDL-C), triglycerides-to-HDL ratio (TG/HDL ratio), visceral adiposity index (VAI), systolic blood pressure (SBP), diastolic blood pressure (DBP) and metabolic syndrome (MetS).

**Table 2:** Characteristics of the population according to BMI z-score and the presence/absence of MetS.

	BMI z-Score < 3		BMI z-Score ≥3			Presence of MetS		Absence of MetS		
	**(*n* = 422)**		**(*n* = 215)**		*p*	(*n* = 79)		(*n* = 558)		*p*
Age	12	(10–13)	11	(9–13)	0.002	9	(8–12)	12	(10–13)	<0.001
Girls	231	(54.7%)	93	(43.3%)	0.006	40	(50.6%)	284	(50.9%)	0.965
Height	1.55	(1.46–1.63)	1.51	(1.44–1.6)	0.026	1.47	(1.39–1.57)	1.54	(1.46–1.62)	<0.001
Weight	68.4	(55.2–81.8)	73.5	(59–91)	<0.001	58	(47.3–81)	71.8	(57.5–84.1)	0.001
BMI	28.4	(25.7–30.9)	31.4	(28.1–35.3)	<0.001	27.8	(24.3–33.2)	29.4	(26.7–31.9)	0.025
BMI z-score	2.7	(2.5–2.9)	3.3	(3.1–3.7)	<0.001	2.9	(2.6–3.4)	2.8	(2.6–3.1)	0.095
WC	92	(84–100)	98	(90–108)	<0.001	88	(82–104)	94	(86–102)	0.019
WHR	0.6	(0.56–0.63)	0.65	(0.61–0.68)	<0.001	0.62	(0.58–0.68)	0.61	(0.58–0.65)	0.153
ABSI	0.0798	(0.0762–0.0833)	0.0805	(0.0771–0.0834)	0.337	0.081	(0.0782–0.0838)	0.0798	(0.0762–0.0833)	0.064
Glucose	85	(80–90)	86	(81–91)	0.041	85	(82–91)	85	(80–90)	0.313
HOMA-IR	2.98	(2.09–4.45)	3.67	(2.51–5.02)	<0.001	4.15	(3.05–5.74)	3.06	(2.11–4.53)	<0.001
HOMA β	259.2	(170.3–359.5)	267.4	(196.2–424.6)	0.037	316.8	(241.1–459.5)	245.2	(171.2–356.6)	<0.001
QUICKI	0.324	(0.307–0.341)	0.315	(0.303–0.332)	<0.001	0.310	(0.297–0.323)	0.323	(0.307–0.341)	<0.001
TyG index	4.39	(4.24–4.57)	4.45	(4.29–4.59)	0.062	4.67	(4.51–4.78)	4.39	(4.24–4.53)	<0.001
TG	77	(57–107)	85	(61–113)	0.105	134	(99–172)	76	(56–100)	<0.001
TC	155	(140–174)	156	(141–181)	0.094	160	(145–182)	155	(139–176)	0.037
HDL-C	46	(40–54)	48	(39–54)	0.622	38	(34–47)	48	(41–54)	<0.001
LDL-C	93	(78–109)	91	(79–106)	0.155	98	(85–119)	92	(77–110)	0.008
TG/HDL ratio	1.7	(1.2–2.4)	1.8	(1.2–2.7)	0.241	3.3	(2–5.2)	1,6	(1.2–2.3)	<0.001
VAI	1.12	(0.75–1.75)	1.19	(0.72–1.79)	0.745	2.36	(1.17–3.52)	1.09	(0.72–1.58)	<0.001
SBP	110	(105–120)	114	(105–120)	0.103	115	(108–123)	110	(105–120)	0.014 *
DBP	70	(60–70)	70	(61–80)	0.002	65	(58–73)	70	(60–71)	0.115
MetS	43	(10.2%)	36	(16.7%)	0.018	-		-		-

Body Mass Index z-score (BMI z-score), Waist Circumference (WC), Waist-to-Height Ratio (WHR), A Body Shape Index (ABSI), Homeostatic Model Assessment Index—Insulin Resistance (HOMA-IR), Homeostatic Model Assessment Index -β (HOMA-β), Quantitative Insulin sensitivity Check Index (QUICKI), Triglyceride Glucose Index (TyG index), Triglycerides (TG), Total cholesterol (TC), High-Density Lipoprotein cholesterol (HDL-C), Low-Density Lipoprotein cholesterol (LDL-C), Triglycerides-to-HDL ratio (TG/HDL ratio), Visceral Adiposity Index (VAI), Systolic blood pressure (SBP), Diastolic blood pressure (DBP), Metabolic Syndrome (MetS). * *p* < 0.050.

**Table 3:** Correlation heatmap of adiposity indices, glyco-metabolic indices, lipids, TG/HDL-C ratio, VAI and blood pressure.

		BMI	BMI z-Score	WC	WHR	ABSI	Glucose	HOMA—IR	HOMA -β	QUICKI	TyG	TG	TC	HDL-C	LDL-C	TG/HDL Ratio	VAI	SBP	DBP	
											index									
BMI	ρ	1.00																		
	*p*																			
BMI z-score	ρ	0.45	1.00																	
	*p*	<0.001																		
WC	ρ	0.82	0.37	1.00																ρ
	*p*	<0.001	<0.001																	−1.00
WHR	ρ	0.50	0.53	0.66	1.00															−0.75
	*p*	<0.001	<0.001	<0.001																−0.50
ABSI	ρ	−0.19	0.11	0.30	0.52	1.00														−0.25
	*p*	<0.001	0.008	<0.001	<0.001															0.00
Glucose	ρ	0.19	0.11	0.14	0.07	−0.09	1.00													0.25
	*p*	<0.001	0.008	0.001	0.101	0.032														0.50
HOMA—IR	ρ	0.40	0.16	0.36	0.22	−0.07	0.43	1.00												0.75
	*p*	<0.001	<0.001	<0.001	<0.001	0.115	<0.001													1.00
HOMA -β	ρ	0.29	0.09	0.27	0.17	−0.03	−0.29	0.70	1.00											
	*p*	<0.001	0.028	<0.001	<0.001	0.502	<0.001	<0.001												
QUICKI	ρ	−0.40	−0.16	−0.35	−0.22	0.07	−0.43	−1.00	−0.70	1.00										
	*p*	<0.001	<0.001	<0.001	<0.001	0.109	<0.001	<0.001	<0.001											
TyG index	ρ	0.15	0.06	0.19	0.18	0.08	0.14	0.44	0.35	−0.44	1.00									
	*p*	<0.001	0.149	<0.001	<0.001	0.051	<0.001	<0.001	<0.001	<0.001										
TG	ρ	0.12	0.04	0.17	0.17	0.09	−0.03	0.36	0.40	−0.36	0.98	1.00								
	*p*	0.002	0.265	<0.001	<0.001	0.025	0.538	<0.001	<0.001	<0.001	<0.001									
TC	ρ	0.07	0.10	0.07	0.14	0.09	0.10	0.13	0.04	−0.14	0.40	0.39	1.00							
	*p*	0.091	0.01	0.065	<0.001	0.024	0.011	0.001	0.303	0.001	<0.001	<0.001								
HDL-C	ρ	−0.15	0.03	−0.20	−0.13	−0.06	0.08	−0.21	−0.30	0.21	−0.34	−0.35	0.19	1.00						
	*p*	<0.001	0.425	<0.001	0.001	0.111	0.049	<0.001	<0.001	<0.001	<0.001	<0.001	<0.001							
LDL-C	ρ	0.08	0.09	0.09	0.13	0.07	0.09	0.17	0.10	−0.18	0.36	0.35	0.88	−0.11	1.00					
	*p*	0.042	0.019	0.032	0.001	0.066	0.019	<0.001	0.011	<0.001	<0.001	<0.001	<0.001	0.007						
TG/HDL ratio	ρ	0.16	0.03	0.21	0.19	0.10	−0.05	0.37	0.43	−0.37	0.91	0.93	0.25	−0.65	0.32	1.00				
	*p*	<0.001	0.475	<0.001	<0.001	0.017	0.228	<0.001	<0.001	<0.001	<0.001	<0.001	<0.001	<0.001	<0.001					
VAI	ρ	0.21	−0.02	0.26	0.23	0.10	−0.06	0.38	0.46	−0.38	0.86	0.88	0.21	−0.63	0.28	0.95	1.00			
	*p*	<0.001	0.716	<0.001	<0.001	0.015	0.116	<0.001	<0.001	<0.001	<0.001	<0.001	<0.001	<0.001	<0.001	<0.001				
SBP	ρ	0.39	0.05	0.31	0.04	−0.21	0.12	0.24	0.19	−0.24	0.12	0.11	−0.02	−0.08	0.02	0.11	0.11	1.00		
	*p*	<0.001	0.254	<0.001	0.288	<0.001	0.004	<0.001	<0.001	<0.001	0.004	0.009	0.71	0.042	0.646	0.007	0.009			
DBP	ρ	0.41	0.22	0.43	0.23	0.06	0.13	0.13	0.06	−0.13	−0.02	−0.04	0.03	−0.04	0.04	−0.01	0.03	0.41	1.00	
	*p*	<0.001	<0.001	<0.001	<0.001	0.166	0.002	0.002	0.13	0.002	0.579	0.376	0.519	0.314	0.307	0.881	0.456	<0.001		

Body Mass Index z-score (BMI z-score), Waist Circumference (WC), Waist-to-Height Ratio (WHR), A Body Shape Index (ABSI), Homeostatic Model Assessment Index—Insulin Resistance (HOMA-IR), Homeostatic Model Assessment Index -β (HOMA-β), Quantitative Insulin sensitivity Check Index (QUICKI), Triglyceride Glucose Index (TyG index), Triglycerides (TG), Total cholesterol (TC), High-Density Lipoprotein cholesterol (HDL-C), Low-Density Lipoprotein cholesterol (LDL-C), Triglycerides-to-HDL ratio (TG/HDL ratio), Visceral Adiposity Index (VAI), Systolic blood pressure (SBP), Diastolic blood pressure (DBP). ρ: Spearman’s correlation coefficient. Color coding according to Spearman correlation coefficient (ρ).

**Table 4:** Logistic regression coefficients (standard errors) of adiposity indices and VAI with the presence of MetS.

	BMI z-Score	WHR z-Score	ABSI z-Score	VAI z-Score
Male sex	0.076 (0.255)	0.022 (0.250)	0.025 (0.252)	−0.684 * (0.298)
Age	−0.299 * (0.065)	−0.319 * (0.066)	−0.307 * (0.066)	−0.413 * (0.076)
BMI z-score	0.416 (0.213)			
WHR z-score		0.319 * (0.098)		
ABSI z-score			0.135 (0.140)	
VAI z-score				1.203 * (0.164)
Costant	0.055 (1.043)	1.531 * (0.721)	1.415 (0.730)	2.698 (0.836)
Cases	637	631	631	628
Pseudo R^2^	0.062	0.076	0.057	0.229
AIC	430	442	455	374

* *p* < 0.050.

## Data Availability

The data presented in this study are available on request from the corresponding author.

## Supplementary Materials

The following are available online at https://www.mdpi.com/2072-6643/13/2/413/s1, Table S1: Characteristics of the subpopulations according to gender and age, Table S2: Heatmap of correlations of VAI with adiposity indices, glyco-metabolic indices, lipids, TG/HDL-C ratio and blood pressure in the subpopulations according to gender and age.
