# Supplementary material for: Visceral Adiposity Index (VAI) in Children and Adolescents with Obesity: No Association with Daily Energy Intake but Promising Tool to Identify Metabolic Syndrome (MetS)
Source: Nutrients. 2021 Jan 28;13(2):413. doi: 10.3390/nu13020413 (PMC7911630; doi:10.3390/nu13020413)
Supplement: Supplementary file 1 [file nutrients-13-00413-s001.pdf]

Supplementary Table S1. Characteristics of the subpopulations according to gender and age.

	Boys aged <10 years (n=57)		Boys aged ≥10 years (n=256)		Girls aged <10 years (n=72)		Girls aged ≥10 years (n=252)	
**Age**	9	(8 - 9)	12	(11 - 13)	9	(8 - 9)	12	(11 - 14)
**Height**	1.39	(1.35 - 1.44)	1.58	(1.5 - 1.67)	1.41	(1.35 - 1.45)	1.57	(1.5 - 1.62)
**Weight**	48	(43.5 - 53)	73.5	(61.7 - 90)	50.1	(45.2 - 55.7)	77	(65.4 - 86.5)
**BMI**	25.2	(23.9 - 27)	29.3	(27.2 - 31.8)	25.3	(24.1 - 27)	31.1	(28.6 - 33.3)
**BMI z-score**	3.1	(2.9 - 3.6)	2.9	(2.6 - 3.1)	2.7	(2.6 - 3.1)	2.8	(2.6 - 3)
**WC**	82	(79 - 86)	97	(91 - 104)	82	(78 - 87)	96	(89 - 103)
**WHR**	0.59	(0.57 - 0.65)	0.62	(0.59 - 0.65)	0.59	(0.56 - 0.63)	0.62	(0.58 - 0.66)
**ABSI**	0.0821	(0.0784 - 0.084)	0.0813	(0.0783 - 0.0837)	0.0797	(0.0777 - 0.0838)	0.078	(0.0741 - 0.0816)
**Glucose**	84	(80 - 89)	86	(81 - 90)	82	(78 - 87)	85	(80 - 91)
**HOMA-IR**	2.09	(1.43 - 3.5)	3.31	(2.3 - 4.69)	2.47	(1.87 - 3.88)	3.66	(2.59 - 5.14)
**HOMA β**	178.6	(120.7 - 247.8)	256.7	(174.3 - 370.4)	239.6	(169.1 - 341)	276.5	(196.3 - 435.3)
**QUICK**	0.34	(0.32 - 0.36)	0.32	(0.31 - 0.34)	0.33	(0.31 - 0.35)	0.32	(0.3 - 0.33)
**TyG index**	4.37	(4.15 - 4.51)	4.44	(4.27 - 4.6)	4.41	(4.25 - 4.63)	4.4	(4.25 - 4.57)
**TG**	71	(48 - 99)	84	(59 - 117)	81	(61 - 126)	79	(58 - 107)
**TC**	154	(142 - 178)	157	(142 - 180)	155	(142 - 177)	154	(136 - 175)
**HDL-C**	52	(46 - 57)	45	(39 - 53)	46	(41 - 54)	47	(40 - 54)
**LDL-C**	92	(77 - 108)	96	(78 - 114)	94	(83 - 111)	91	(77 - 110)
**TG/HDL ratio**	1.48	(0.95 - 2.06)	1.81	(1.16 - 2.71)	1.72	(1.25 - 3.1)	1.69	(1.19 - 2.35)
**VAI**	0.77	(0.48 - 1.09)	1.05	(0.66 - 1.61)	1.36	(0.93 - 2.2)	1.37	(0.95 - 1.96)
**SBP**	105	(100 - 110)	115	(110 - 120)	105	(99 - 113)	114	(109 - 120)
**DBP**	60	(58 - 70)	70	(60 - 77)	60	(57 - 70)	70	(63 - 75)
**MetS**	18	(31.6%)	21	(8.2%)	26	(36.1%)	14	(5.6%)

Body Mass Index z-score (BMI z-score), Waist Circumference (WC), Waist-to-Height Ratio (WHR), A Body Shape Index (ABSI), Homeostatic Model Assessment Index – Insulin Resistance (HOMA-IR), Homeostatic Model Assessment Index -β (HOMA-β), Quantitative Insulin sensitivity Check Index (QUICKI), Triglyceride Glucose Index (TyG index), Triglycerides (TG), Total cholesterol (TC), High-Density Lipoprotein cholesterol (HDL-C), Low-Density Lipoprotein cholesterol (LDL-C), Triglycerides-to-HDL ratio (TG/HDL ratio), Visceral Adiposity Index (VAI), Systolic blood pressure (SBP), Diastolic blood pressure (DBP), Metabolic Syndrome (MetS).

Supplementary Table S2. Heatmap of correlations of VAI with adiposity indices, glyco-metabolic indices, lipids, TG/HDL-C ratio and blood pressure in the subpopulations according to gender and age.

		VAI						
		**Whole cohort**	**Boys**	**Boys <10 years**	**Boys ≥10 years**	**Girls**	**Girls <10 years**	**Girls ≥10 years**
BMI	ρ	0.21	0.32	0.14	0.26	0.07	-0.05	0.09
	P	<0.001	<0.001	0.311	<0.001	0.237	0.654	0.145
BMI z-score	ρ	-0.02	0.01	0.16	0.07	0.06	-0.13	0.12
	P	0.716	0.798	0.246	0.277	0.285	0.270	0.052
WC	ρ	0.26	0.37	0.27	0.32	0.22	0.14	0.28
	P	<0.001	<0.001	0.039	<0.001	<0.001	0.246	<0.001
WHR	ρ	0.23	0.21	0.30	0.18	0.28	0.18	0.32
	P	<0.001	<0.001	0.026	0.004	<0.001	0.137	<0.001
ABSI	ρ	0.10	0.08	0.23	0.05	0.28	0.28	0.30
	P	0.015	0.182	0.088	0.383	<0.001	0.020	<0.001
Glycemia	ρ	-0.06	0.03	0.02	0.00	-0.12	-0.06	-0.13
	P	0.116	0.634	0.857	0.972	0.039	0.631	0.044
HOMA -IR	ρ	0.38	0.47	0.35	0.44	0.28	0.36	0.29
	P	<0.001	<0.001	0.008	<0.001	<0.001	0.003	<0.001
HOMA -β	ρ	0.46	0.47	0.40	0.44	0.42	0.46	0.41
	P	<0.001	<0.001	0.002	<0.001	<0.001	<0.001	<0.001
QUICK	ρ	-0.38	-0.46	-0.36	-0.44	-0.29	-0.36	-0.29
	P	<0.001	<0.001	0.007	<0.001	<0.001	0.003	<0.001
TyG index	ρ	0.86	0.92	0.92	0.92	0.88	0.87	0.88
	P	<0.001	<0.001	<0.001	<0.001	<0.001	<0.001	<0.001
TG	ρ	0.88	0.93	0.94	0.93	0.91	0.91	0.91
	P	<0.001	<0.001	<0.001	<0.001	<0.001	<0.001	<0.001
TC	ρ	0.21	0.23	0.32	0.23	0.26	0.09	0.31
	P	<0.001	<0.001	0.017	<0.001	<0.001	0.467	<0.001
HDL-C	ρ	-0.63	-0.70	-0.65	-0.67	-0.61	-0.74	-0.56
	P	<0.001	<0.001	<0.001	<0.001	<0.001	<0.001	<0.001
LDL-C	ρ	0.28	0.28	0.41	0.25	0.35	0.27	0.37
	P	<0.001	<0.001	0.001	<0.001	<0.001	0.022	<0.001
TG/HDL ratio	ρ	0.95	0.99	0.99	0.99	0.99	0.99	0.99
	P	<0.001	<0.001	<0.001	<0.001	<0.001	<0.001	<0.001
SBP	ρ	0.11	0.20	0.25	0.13	0.05	0.26	0.00
	P	0.009	0.001	0.066	0.045	0.369	0.040	0.941
DBP	ρ	0.03	0.03	-0.09	-0.01	0.03	0.06	0.02
	P	0.456	0.566	0.526	0.902	0.629	0.657	0.766

ρ
-1.00
-0.75
-0.50
-0.25
0.00
0.25
0.50
0.75
1.00

Body Mass Index z-score (BMI z-score), Waist Circumference (WC), Waist-to-Height Ratio (WHR), A Body Shape Index (ABSI), Homeostatic Model Assessment Index – Insulin Resistance (HOMA-IR), Homeostatic Model Assessment Index - β (HOMA-β), Quantitative Insulin sensitivity Check Index (QUICKI), Triglyceride Glucose Index (TyG index), Triglycerides (TG), Total cholesterol (TC), High-Density Lipoprotein cholesterol (HDL-C), Low-Density Lipoprotein cholesterol (LDL-C), Triglycerides-to-HDL ratio (TG/HDL ratio), Visceral Adiposity Index (VAI), Systolic blood pressure (SBP), Diastolic blood pressure (DBP). Color coding according to Spearman correlation coefficient (ρ).
